# Optimized CatBoost machine learning (OCML) for DDoS detection in cloud virtual machines with time-series and adversarial robustness

**DOI:** 10.1038/s41598-025-33851-5

**Published:** 2026-01-15

**Authors:** Hadeer Samy, Ayman M. Bahaa-Eldin, Mohamed A. Sobh, Ayman Taha

**Affiliations:** 1 Electrical Engineering Department, Higher Technological Institute, Tenth of Ramadan City, Al-Sharqia, Egypt; 2 Faculty of Engineering, Computer and System Engineering Department, Ain Shams University, Cairo, Egypt; 3 Elsewedy University of Technology, Tenth of Ramadan City, Al-Sharqia, Egypt

**Keywords:** OCML, DDoS, Optuna, Catboost, SHAP, Adversarial attacks, Engineering, Mathematics and computing

## Abstract

Distributed Denial of Service (DDoS) attacks represent one of the most strategically executed and severe threats in cloud computing, often leading to substantial data loss and significant financial damage for both cloud service providers and their users. Numerous studies have been conducted to enhance cloud security against such attacks through the application of machine learning techniques. This paper implements the Optimized Catboost machine learning algorithm (OCML) with hyperparameter optimization using Optuna to achieve efficient training. Feature selection was conducted using the SHAP (SHapley Additive exPlanations) method, as the dataset contains over 80 features. The proposed model achieved an accuracy of 99.2% in detecting Distributed Denial of Service (DDoS) attacks in cloud virtual machines (VMs), enabling the system to filter out malicious jobs and allocate resources efficiently. The CICIDS 2019 dataset was used as the benchmark for evaluation. Furthermore, the robustness of the proposed model was assessed using adversarial attacks, specifically the Fast Gradient Sign Method (FGSM), the Carlini-Wagner (CW) attack, and Projected Gradient Descent (PGD). The Catboost model achieves accuracies against these attacks 97%, 80% and 71% respectively. In addition, the robustness against time series network traffic attacks using pulse wave, random burst, and slow ramp achieves 80%, 83% and 77% respectively.

## Introduction

Cloud computing represents an advanced technological paradigm that enables the delivery of computing services to end users referred to as cloud service consumers through a variety of capabilities, including on-demand resource provisioning, pay-per-use pricing, enhanced data security, remote accessibility, and high service availability^1^. This paradigm is typically classified into three primary service models. Infrastructure as a Service (IaaS) provides configurable foundational resources such as servers, networking components, storage, and computing capabilities. Platform as a Service (PaaS) offers development environments, frameworks, and tools for building cloud-based applications. Software as a Service (SaaS) grants end users access to cloud-hosted applications tailored to their specific requirements^2,3^.

Despite its advantages, cloud computing remains susceptible to diverse security threats that compromise the privacy, integrity, and confidentiality of stored data. Given that data constitutes a critical asset, ensuring its security is a fundamental obligation of cloud service providers, as specified in service-level agreements. To address these concerns, providers must implement robust and comprehensive defense mechanisms. Nevertheless, all three service models remain vulnerable to a range of cyberattacks^4,5^.

**Fig. 1:**
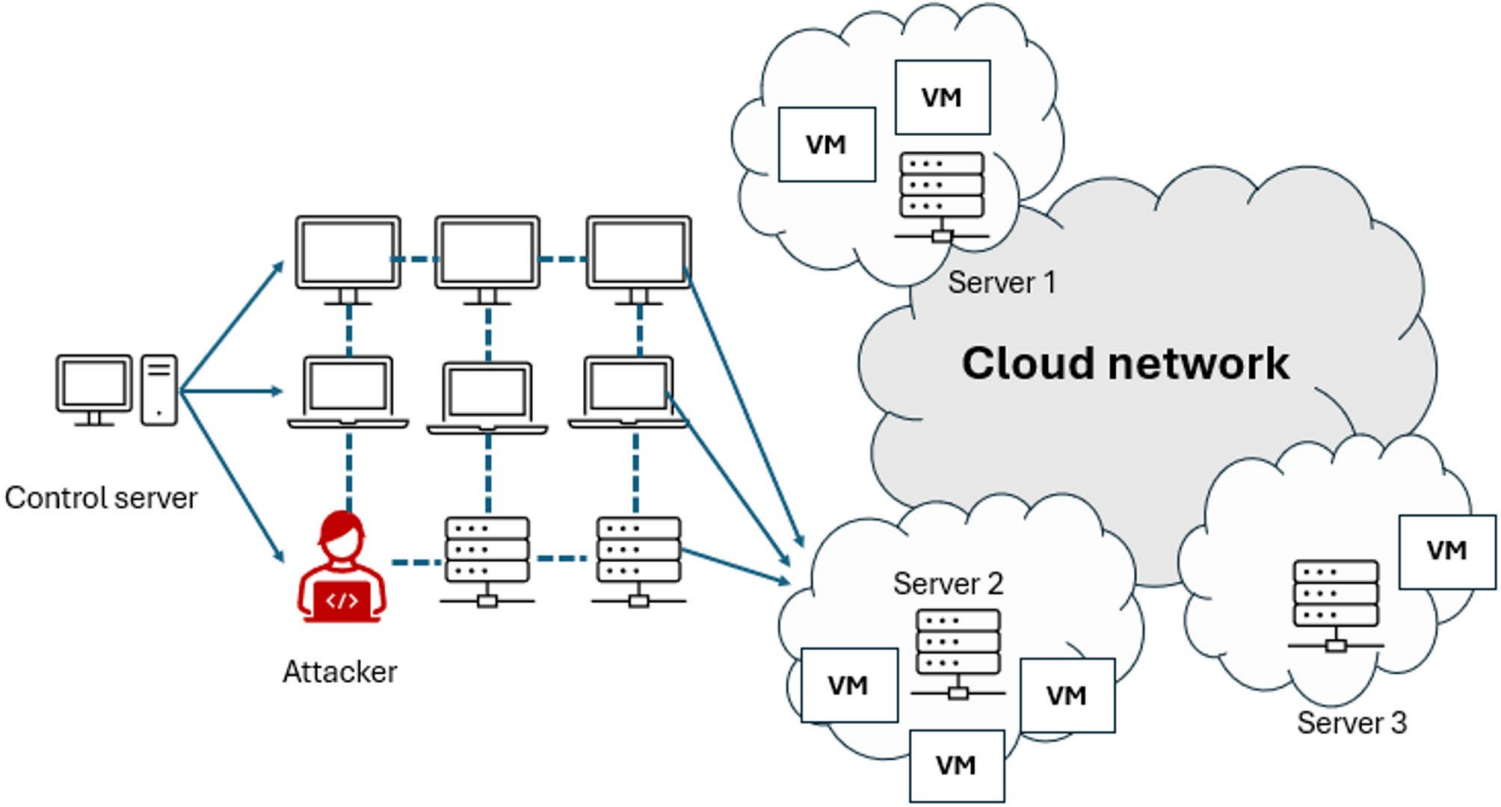
DDoS architecture in cloud computing^7^.

Among these threats, Distributed Denial of Service (DDoS) attacks have escalated considerably in recent years, posing severe challenges to the stability of cloud ecosystems. A DDoS attack overwhelms the target application’s underlying server with a large volume of malicious traffic, depleting its computational resources and obstructing its ability to process legitimate requests. This leads to significant service disruptions and reduced availability^6^. Figure 1 illustrates a Distributed Denial of Service (DDoS) attack architecture targeting cloud-based virtualized infrastructures. In this scenario, an attacker utilizes a control server to orchestrate a botnet that directs coordinated traffic toward multiple virtual machines (VMs) hosted on different cloud servers. The large-scale and distributed nature of this attack overwhelms shared cloud resources, causing rapid exhaustion of CPU, memory, and network bandwidth, which in turn degrades service performance. Moreover, such attacks exploit inherent virtualization vulnerabilities, where resource contention among co-located VMs amplifies the impact. In more advanced cases, adversaries may attempt to compromise the hypervisor layer, threatening the isolation between VMs. This architecture underscores the growing susceptibility of cloud environments to multi-vector DDoS threats and highlights the necessity for adaptive, multi-layered defense mechanisms capable of detecting and mitigating attacks at both the VM and hypervisor levels.

Cloud VMs face multi-vector DDoS attacks combining volumetric, protocol, and application-layer floods, often targeting resource exhaustion and hypervisor communication channels. These evolving attack vectors complicate detection due to elastic resource scaling and inter VM traffic amplification, multi-vector forms that simultaneously target multiple layers of the network stack^8,9^. These complex threats exploit virtualization vulnerabilities, particularly by overwhelming the limited resources of virtual machines (VMs) such as CPU, memory, and I/O capacity, resulting in rapid resource depletion and service degradation. In addition, advanced persistent threats may extend their focus to compromising the hypervisor layer, rendering detection mechanisms confined to the VM level inadequate^10^. This evolution in attack behavior necessitates a paradigm shift from static, signature-based defenses toward adaptive, behavior-driven detection models, highlighting the urgent need for intelligent and resilient security frameworks capable of countering dynamic and diverse adversarial strategies.

However, the increasing reliance on cloud services has also made them a prime target for cyberattacks, particularly Distributed Denial of Service (DDoS) attacks. These attacks aim to overwhelm virtual machines (VMs) with malicious traffic, disrupting services and causing significant financial and reputational damage. As cloud environments continue to grow in complexity, traditional security measures often fall short in detecting and mitigating such sophisticated attacks. This underscores the need for advanced, intelligent solutions that can predict and prevent DDoS attacks while ensuring efficient resource allocation in cloud infrastructures^11^.

Despite the growing application of machine learning to DDoS detection, most existing models focus solely on achieving high accuracy on static datasets while overlooking adversarial robustness and temporal variability. Such limitations hinder their effectiveness against evolving, multi-vector cloud attacks that exploit VM-level vulnerabilities such as CPU resource exhaustion, hypervisor overload, and bandwidth saturation.

To address these challenges, this study proposes an Optimized CatBoost Machine Learning (OCML) framework that integrates three complementary mechanisms: (1) Optuna-based Bayesian hyperparameter optimization for efficient parameter tuning, (2) SHAP-driven feature selection to enhance interpretability and dimensionality reduction, and (3) adversarial training to strengthen resilience against gradient-based perturbations.

Hypothesis: We hypothesize that the OCML framework, integrating explainable feature selection, hyperparameter optimization, and adversarial training into a unified CatBoost pipeline, will significantly improve both detection accuracy and robustness against evolving DDoS attack patterns in cloud virtual machines (VMs).

### Research gap and motivation

Recent advances in DDoS detection using deep and ensemble learning have achieved high detection rates (≈ 99%) on benchmark datasets such as CICIDS2017 and CICDDoS2019. However, three critical gaps persist:

1. Limited adversarial robustness: Most studies lack evaluation against strong white-box attacks (FGSM, CW, PGD).
2. Limited temporal adaptability: Existing detectors are trained on static data, failing to identify evolving time-series attack dynamics (pulse wave, random burst).
3. Lack of optimization transparency: Manual or grid-based hyperparameter tuning and unexplainable feature selection restrict generalizability.
4. Much research has focused on hardening image classifiers; the robustness of tree-based models like CatBoost is less explored.

The OCML framework bridges these gaps by systematically optimizing CatBoost parameters using Bayesian search, selecting interpretable features with SHAP, and validating resilience under adversarial and dynamic time-series scenarios.

## Related works

### DDoS intrusion detection by machine learning

This section reviews existing studies on Distributed Denial of Service (DDoS) attack detection in cloud virtual machine (VM) environments using machine learning (ML) techniques. It summarizes the methodologies, datasets, and key findings of prior research to identify existing gaps addressed in this study, with a concise overview presented in Table 1.

Shine Rajesh^12^ employed machine learning (ML) techniques for anomaly detection using a customized CERT dataset, comparing ensemble models such as LightGBM, XGBoost, AdaBoost, and Random Forest. Similarly, Vanitha^13^ proposed an automated insider threat mitigation framework that reduces false positives and enhances incident response efficiency, emphasizing ensemble learning’s role in cloud security. While Premkumar Reddy and Shang, Yongqiang^14,15^ utilized the NSL-KDD dataset with Learning Vector Quantization (LVQ) and Principal Component Analysis (PCA) for dimensionality reduction, classifying features using Decision Tree, Naïve Bayes, and SVM to detect DDoS patterns. Similarly, Himanshu Setiaa^16^ further introduced a VANET cloud-based framework that applies ML for DDoS detection through network flow analysis.

Reddy, Sai Sindhu Theja^17^ developed a secure SaaS framework integrating a Deep Belief Network (DBN) optimized via the MFSLnO algorithm and a bait-based mitigation mechanism for attack isolation. Dasari, Sandeep. Dasari, Sandeep^18^ proposed a hierarchical ML-based hyperparameter optimization framework using the CICIDS 2017 dataset, combining preprocessing techniques such as SMOTE and LASSO with LightGBM, XGBoost, and CatBoost models. Furthermore, Hanaa Attou^19^ introduced a cloud-based intrusion detection model using Random Forest and feature engineering, validated on the Bot-IoT and NSL-KDD datasets. Sanjalawe, Yousef^20^ designed a hybrid CNN-LSTM intrusion detection system with ensemble feature selection (PSO, GWO, KH, WOA), achieving 98.3% accuracy on CICIDS 2017.

Bagyalakshmi^21^ combined LVQ and PCA feature selection with NB, SVM, and DT classifiers on NSL-KDD, identifying LVQ-DT as the best performer. Mohamed Ouhssini^22^ proposed DeepDefend, a real-time DDoS detection framework integrating CNN–LSTM–Transformer networks with a genetic algorithm for feature selection, achieving high precision on CIDDS-001. SARAH NAIEM^23^ improved Gaussian Naïve Bayes through hybrid feature selection (Pearson, Mutual Information, Chi-squared) and preprocessing (SMOTE, mode–mean substitution), enhancing accuracy and precision. Gopal Singh Kushwah^24^ introduced a Self-adaptive Evolutionary Extreme Learning Machine (SaE-ELM) for dynamic operator and hidden layer optimization, while Manisankar Sannigrahi^25^ applied Bayesian-optimized and hybrid Random Forest models to KDD99 and NSL-KDD, achieving robust classification results.

Recent studies developed by Sumathi, S.Rajesh, R.Lim, Sangsoon^26^ have introduced hybrid machine learning and deep learning models to improve intrusion detection accuracy in cloud and network environments. An ANN-based GBS framework combining GWO, BPN, and SOM achieved high accuracy on UNSW-NB15 by integrating correlation-based feature selection, 10-fold cross-validation, and GWO-driven hyperparameter tuning. Similarly^27^, an SVM-based IDS optimized using a hybrid HHO-PSO algorithm and validated on NSL-KDD outperformed classical classifiers across multiple performance metrics. Another study^28^ combined an LSTM with an autoencoder–decoder architecture, using hybrid HHO-PSO optimization for parameter tuning and feature selection, yielding superior detection performance compared with existing deep learning models.

Finally, Ibtihal AlSaleh and Aida Al-Samawi^29^ developed a Bayesian-based CNN (BaysCNN) using PCA and the CICDDoS2019 dataset for DDoS detection. In addition, Abdul Salam^30^ proposed a Machine Learning–Assisted Cloud Computing Model (ML-CCM) that integrates big data analytics to enhance cloud security and data transmission.

### Adversarial machine learning (AML)

Ensemble learning integrates multiple machine learning (ML) models to enhance classification performance and robustness against adversarial attacks^31^.

Apruzzese et al.^32^ introduced the FGMD framework, which employs feature grouping and ensemble LSTM models to detect malicious IoT traffic and resist evasion attacks by generating realistic adversarial samples. Evaluations on MedBloT and IoTID datasets demonstrated superior accuracy and robustness compared to baseline models. Similarly, Alotaibi^33^ developed a CNN-based intrusion detection system (IDS) that maintained high accuracy under normal conditions but exhibited significant performance degradation under adversarial attacks, mitigated through the APE_GAN + + defense mechanism. In parallel, Holla^34^ examined ML-based IDSs on the CSE-CIC-IDS2018 dataset, revealing substantial accuracy losses under FGSM, PGD, and DeepFool attacks, and proposed a dual defense strategy combining adversarial training with SHAP-based feature selection to enhance model resilience.

### Intrusion detection based on time series

This step is crucial for identifying relationships among time-series variables and detecting anomalous patterns preceding potential security breaches. Nachaat Mohamed^35^ conducted a comprehensive analysis of adversarial defense strategies to enhance AI resilience against attacks and emphasized the role of federated learning in enabling privacy-preserving, decentralized threat intelligence. Similarly, Farman Ullah^36^ proposed a BiLSTM-based framework for forecasting cloud VM resource utilization using multivariate time-series data, demonstrating high predictive accuracy across key metrics such as CPU, memory, disk I/O, and network throughput.

A novel intrusion detection approach for cloud computing environments based on time series analysis was introduced by Abdel-Rahman Al-Ghuwairi^37^. proposed a time-series–based intrusion detection approach for cloud environments that integrates anomaly detection, stationarity, and causality tests for collaborative feature selection, effectively reducing input features from 70 to 10 and enhancing prediction accuracy while minimizing execution time. Similarly, Mateusz Smendowski^38^ introduced the Multi-Time Series Forecasting System (MSFS) with a Hybrid Ensemble Anomaly Detection Algorithm (HEADA) and a Similarity-based Time-Series Grouping (STG) method, improving scalability, interpretability, and resource optimization in large-scale cloud infrastructures.

Marina Krotofil^39^ investigated optimal timing strategies for launching DoS attacks in the Tennessee Eastman process, revealing that attack success depends on process dynamics and timing, thereby identifying critical sensors for protection and timely incident response. However, Moses blessing^40^ evaluated time-series forecasting models such as ARIMA, exponential smoothing, and machine learning techniques for predicting cyberattacks from historical data, demonstrating improved early detection and reduced false positives, while emphasizing the need for real-time data integration and advanced ML enhancements.

**Table 1:** Comparison of reviewed literature.

Author (no.)	Year	Methodology	Result	Limitations
D Shine Rajesh^12^	2024	Ensemble models on the CERT dataset using LightGBM, XGBoost, AdaBoost, RF	LightGBM achieved the highest accuracy (97%), better than XGBoost (88.27%), AdaBoost (88%), and RF (86%)	The dataset limitation, which may restrict generalizability, and only the accuracy dependence
M. Vanitha^13^	2024	A framework using ensemble learning for insider threat detection	Reduced false positives and enhanced response effectiveness	Neglect of emerging insider threat patterns; no focus on adversarial resilience.
Premkumar Reddy et al.^14,15^	2023, 2024	NSL-KDD dataset with LVQ, PCA for feature selection, classifiers: DT, NB, SVM	Combined feature selection and ML improve DDoS detection.	Lacks real-time detection and robustness due to single-algorithm reliance.
Himanshu Setiaa^16^	2024	VANET Cloud with ML classification and predictive analytics	Achieved 99.59% accuracy	Need for hyperparameter tuning,
Reddy, Sai Sindhu Theja^17^	2022	SaaS framework with Deep Belief Network (DBN) and MFSLnO optimization	Neutralizes attack nodes with minimal disruption	Lacks prevention mechanisms and employs limited evaluation metrics.
Dasari, Sandeep^18^	2024	CICIDS2017 intrusion detection using ML with Min-Max scaling, SMOTE, LASSO, and boosting models.	LGBM achieved 99.77% accuracy for DDoS detection	Focuses solely on accuracy, omitting other metrics and adversarial resilience.
Hanaa Attou^19^	2023	RF classifier with feature engineering on Bot-IoT and NSL-KDD datasets	Achieved 98.3% (Bot-IoT) and 99.99% (NSL-KDD) accuracy	Uses only two features, which may restrict the effectiveness of the detection capability
Sanjalawe, Yousef^20^	2023	CNN-LSTM with ensemble feature selection (PSO, GWO, KH, WOA) on CICIDS 2017	Achieved up to 98.3% accuracy	not validated on streaming data for real-time detection.
Mohamed Ouhssini^22^	2024	DeepDefend: CNN-LSTM-Transformer + GA feature selection on CIDDS-001	High precision in real-time DDoS detection	No real-world deployment, and scalability under unpredictable traffic remains untested.
Sarah Naiem^23^	2023	Gaussian NB with hybrid feature selection (Pearson, MI, Chi-squared), preprocessing with SMOTE	2% gain for the MI model; 1.5% overall accuracy and precision improvement	Compared to other classifiers, GNB performs poorly and fails with unseen features.
Manisankar Sannigrahi^25^	2024	BO-RF for binary, hybrid LR-RF for multiclass classification on KDD99 and NSL-KDD	Strong performance across accuracy, precision, recall, and F1-score	The datasets are old and may not fully represent modern network traffic or attack patterns.
Ibtihal AlSaleh, Aida Al-Samawi^29^	2024	BaysCNN and BaysFusCNN using CICDDoS2019 and PCA	Achieved 99.66% (BaysCNN) and 99.79% (BaysFusCNN) accuracy	not explicitly tested in real-time cloud environments, Performance evaluation limitation

### Main contributions

The key contributions of this study are summarized as follows:

1. Hybrid OCML framework: Developed a unified model integrating CatBoost, Optuna optimization, and SHAP feature selection for DDoS detection in cloud VMs.
2. Quantified performance improvement: Achieved 99.2% baseline accuracy, representing a + 1.5% improvement over the best prior CatBoost model, while maintaining a low false-positive rate of 0.5%.
3. Robustness verification: Demonstrated 84.4% mean accuracy under FGSM, CW, and PGD attacks, reducing the attack success rate by 17.3% compared with the untuned baseline.
4. Generalization to dynamic attacks: Validated model performance on time-series traffic patterns (Pulse Wave, Random Burst, Slow Ramp) with F1-scores between 0.77 and 0.83, confirming adaptability to evolving attack behaviors.
5. Ablation and statistical analysis: The Full_OCML model achieved statistically significant improvements over all ablated variants (*p* < 0.001), increasing clean accuracy by up to 9.4%, adversarial accuracy by 57.9%, and reducing robustness drop by 72.7%. Effect sizes ranged from medium to large, and feature efficiency improved using only 10 features, demonstrating the substantial contribution of each OCML component to both accuracy and robustness.

## System model and problem statement

**Fig. 2:**
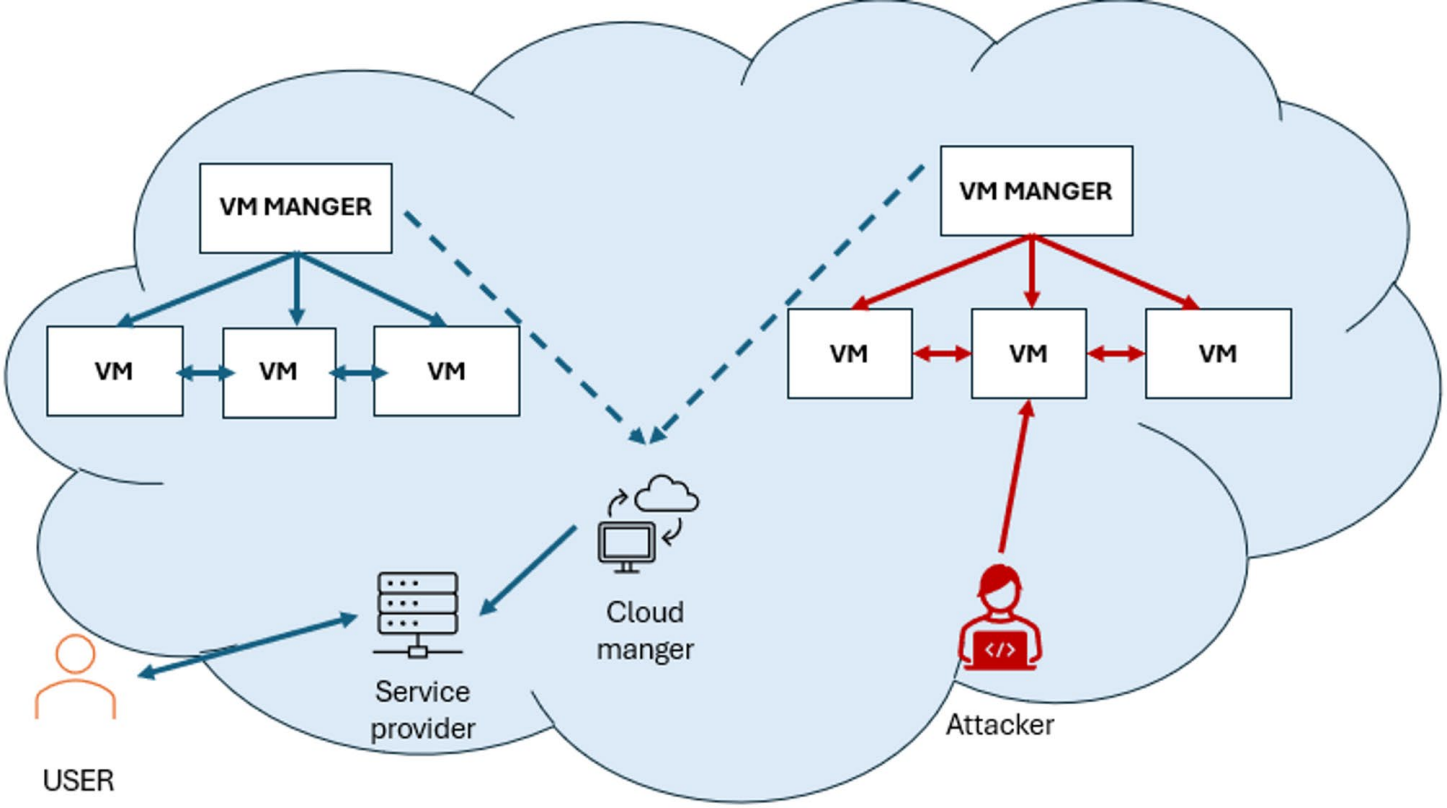
The System model and problem statement.

Virtual Machines (VMs) have been adopted to manage data efficiently. However, they are susceptible to security threats, particularly during the virtualization process. Consequently, implementing robust security mechanisms is a critical priority to mitigate these vulnerabilities. The necessity for securing cloud-based VMs is therefore outlined as follows; If we have a set of VMs connected and each performs a specific task, in such a scenario, if an attacker compromises the address of a single virtual machine (VM), it can disrupt the entire communication process among interconnected VMs, potentially resulting in system instability or damage to the affected VM. Additionally, malicious activities can lead to critical resource exhaustion, including loss of VM storage, further impacting the overall system performance and reliability. Figure 2 shows the system model and problem statement.

Implementing a security module for cloud virtual machines (VMs) is crucial to ensuring data privacy, especially given the advanced capabilities of cloud applications to handle multi-user data simultaneously. These complexities, along with rapid technological advancements, have driven this research toward enhancing security in cloud computing environments. The primary goal of this study is to safeguard interconnected VMs within the cloud paradigm, ensuring the confidentiality of user data. Furthermore, the effectiveness of the proposed security mechanism is evaluated through a use case that simulates a dynamic cloud environment with evolving DDoS attack patterns to assess the algorithm’s detection capabilities.

## Proposed methodology

**Fig. 3:**
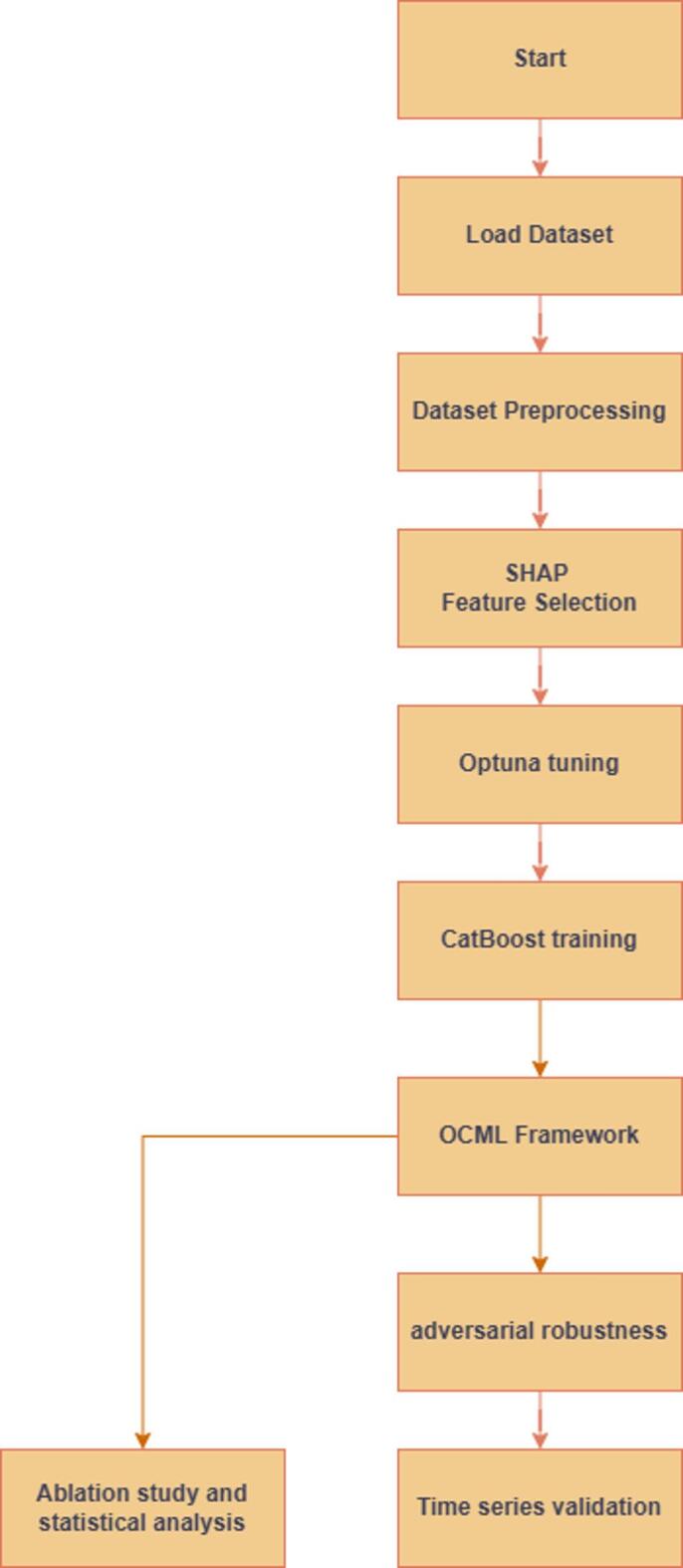
OCML methodological workflow.

The overall workflow of the proposed OCML system is depicted in Fig. 3. The pipeline begins with dataset preprocessing and feature engineering, followed by SHAP-based feature selection to extract the most influential traffic indicators. The CatBoost classifier is then optimized via Bayesian search using the Optuna framework to identify optimal hyperparameter combinations. The optimized model undergoes adversarial training with the PGD method to improve resilience, and its robustness is tested against three adversarial attacks (FGSM, CW, PGD). Finally, the hardened model is evaluated under dynamic time-series attack scenarios simulating realistic DDoS patterns in a virtualized cloud testbed.

### Dataset preprocessing

The study employs the CIC-IDS2019 dataset, a subset containing labeled DDoS attack traffic. The preprocessing pipeline begins with precise data cleaning, removing noninformative columns such as FlowID and Timestamp, while handling missing values and infinite numbers through imputation. The class imbalance was handled not through oversampling techniques like SMOTE, but by leveraging the native capabilities of the CatBoost algorithm, which uses a weighted loss function to mitigate the bias towards the majority class. This approach is preferred as it avoids the potential for overfitting introduced by synthetic data points.

### Feature reduction by SHAP

To identify the most influential predictors for DDoS detection, SHAP (SHapley Additive exPlanations) values were computed across all 80 candidate features. Features were ranked by their mean absolute SHAP values to quantify their contribution to the model’s output. A critical inflection point was observed in the ranked SHAP curve after the first ten features, which indicated a rapid decline in marginal importance. This data-driven threshold was selected to define the final feature set, resulting in the retention of the top 10 SHAP-ranked features. This step effectively reduced the feature space by 87.5%, significantly decreasing computational overhead while preserving the most informative traffic characteristics.

### Catboost development and optimization

The detection system utilizes a Catboost classifier, selected for its superior handling of categorical features and robustness to noisy data, and for overcoming the missing values in large datasets. The model architecture employs gradient boosted decision trees with ordered boosting, which prevents target leakage during training. The experimental design was precisely configured to ensure both the robustness and reproducibility of the Optimized CatBoost Machine Learning (OCML) framework. The CIC-IDS2019 dataset was partitioned using a standard 70% training and 30% testing split. This ratio was selected to provide a sufficiently large and representative sample for model training while reserving a substantial, unseen portion for unbiased performance evaluation. For the hyperparameter optimization phase, the Optuna framework was configured to explore a defined search space for the CatBoost model, as illustrated in Table 3. The 100-trial hyperparameter optimization, leveraging Optuna’s “MedianPruner” to prune unpromising trials early based on intermediate performance metrics against the median of previous trials, demonstrated high efficiency by identifying the optimal parameter set within the initial 10 trials.

Finally, the model’s performance was assessed using a comprehensive suite of metrics—Accuracy, Precision, Recall, F1-score, and Area Under the Curve (AUC). This multi-metric approach is critical for evaluating classification models on highly imbalanced datasets, such as the CIC-IDS2019, where the F1-score and AUC provide a more reliable measure of performance than simple accuracy, particularly in minimizing costly false negatives (missed attacks).

### Cloud environment simulation and attack modeling

The cloud infrastructure is simulated as a dynamic cluster of virtual machines (VMs), each configured with heterogeneous resources (4 vCPUs, 16GB RAM, 100 Mbps bandwidth). Real-time monitoring tracks three key utilization metrics: CPU load calculated as the percentage of allocated vCPUs, memory usage as a fraction of allocated GB, and network congestion as the ratio of current traffic to maximum bandwidth.

### White box adversarial attacks

The methodology for evaluating adversarial robustness involved training a surrogate neural network model, a Sequential Keras model. This surrogate model was then used to generate adversarial examples via three distinct attack methods. The defensive strategy employed in this study was adversarial training using the Projected Gradient Descent (PGD) attack, a strong iterative method considered a standard benchmark for generating adversarial examples. The model was trained for 5 epochs, where each training batch was perturbed using 10 PGD Iterations. Adversarial training is computationally expensive due to the need for attack generation in every batch across all epochs. The Projected Gradient Descent (PGD-10) offers an effective balance by generating sufficiently strong adversarial examples to promote genuine robust feature learning and avoid the issue of gradient masking, while maintaining computational feasibility during training. PGD attack constrained by norm of ε = 0.1. This value is widely accepted in the adversarial machine learning literature as a standard, strong perturbation magnitude that tests the model’s resilience against a significant, yet realistic, level of attack noise. To rigorously evaluate the resulting model’s robustness, a comprehensive white-box assessment was conducted. The evaluation suite consisted of three distinct attacks: (i) the single-step FGSM attack, representing a baseline threat; (ii) the optimization-based C&W L2 attack, chosen to detect potential gradient masking and evaluate robustness against different threat norms; and (iii) a significantly stronger PGD attack with 20 iterations, designed to determine the model’s performance limit under the same threat model used for training. All evaluation attacks were performed under a uniform perturbation budget of ε = 0.1 to ensure a fair and direct comparison. All system parameters are illustrated in Table 2.

**Table 2:** Simulation parameters.

Operating system	Windows 10
Architecture	X64
Dataset	CIC-DDoS2019 dataset “updated version”
CPU	Intel(R) core(TM) i5 5300U CPU @ 2.30 GHz
Storage capacity	8 GB

### Time series DDOS attack detection

To evaluate the efficacy of the trained Catboost model in detecting DDoS attacks within a time series, an experimental environment comprising five virtual machines (VMs) will be established. Two VMs will operate under normal, unattacked conditions, serving as a baseline for resource utilization. The remaining three VMs will be subjected to simulated DDoS attacks, incorporating three distinct attack patterns: pulse wave, random burst, and slow ramp, designed to mimic realistic attack behaviors. During these simulations, critical resource utilization metrics, including CPU, memory, and network traffic, will be continuously monitored and collected from all five VMs. The collected time series data, encompassing both normal and attack-induced resource fluctuations, will then be fed into the trained Catboost model. The primary objective of this methodology is to assess the model’s ability to accurately identify and classify the spikes and anomalies in resource utilization data that correspond to the simulated DDoS attack events, thereby validating its real-world detection capabilities.

### Experimental setup and ablation study

To quantify the individual contribution of each component of the proposed OCML framework, namely, SHAP-based feature selection, Optuna hyperparameter optimization, and the integrated training pipeline, an ablation study was conducted using four model variants: (i) Baseline CatBoost (no SHAP, no Optuna), (ii) Without_SHAP, (iii) Without_Optuna, and (iv) the Full OCML model. Each configuration was evaluated using clean test data and adversarially perturbed data generated using the same attack budget (ε = 0.1) to ensure comparability. Table 5 summarizes the results.

### Statistical validation and significance testing

A statistical analysis was performed to assess the contributions of SHAP feature selection and Optuna hyperparameter optimization within the OCML framework, using four model variants (Baseline, Without_SHAP, Without_Optuna, and Full_OCML). Clean Accuracy and Adversarial Accuracy were analyzed as continuous dependent variables under identical experimental conditions. Overall performance differences were evaluated using One-Way ANOVA, followed by Tukey’s HSD post-hoc test to control the family-wise error rate (α = 0.05). Effect sizes were quantified using Cohen’s d for pairwise comparisons and Eta Squared (η^2^) for ANOVA. These tests were selected because ANOVA determines whether statistically significant differences exist between groups, while the post hoc analysis identifies the specific group pairs that differ significantly.

## Results and discussion

Initially, the VM is designed to perform specific tasks on our dataset within the Jupyter environment. The proposed model includes an updated data sharing mechanism within the replica framework, ensuring secure and efficient communication. The system continuously monitors, detects, and mitigates malicious attacks, in that way enhancing VM security. Following attack detection and mitigation, the virtual machine is dynamically allocated to facilitate the secure sharing of user-requested data. Consequently, data transmission occurs through a protected pathway, ensuring confidentiality and integrity.

The primary objective of this model is to protect virtual machines from DDOS activities while optimizing execution time. Optuna is employed to optimize hyperparameter performance, fine-tuning the model parameters for improved efficiency. Additionally, the execution parameters of Catboost are systematically analyzed, as detailed in Table 3. Then test the model with adversarial attacks through case studies A and B.

**Table 3:** Optuna hyperparameters for the catboost training model.

Parameters	Ranges
Iterations	[100–1000]
Depth	[4–10]
learning_rate	[0.01–0.2]
l2_leaf_reg	[1–20]

**Fig. 4:**
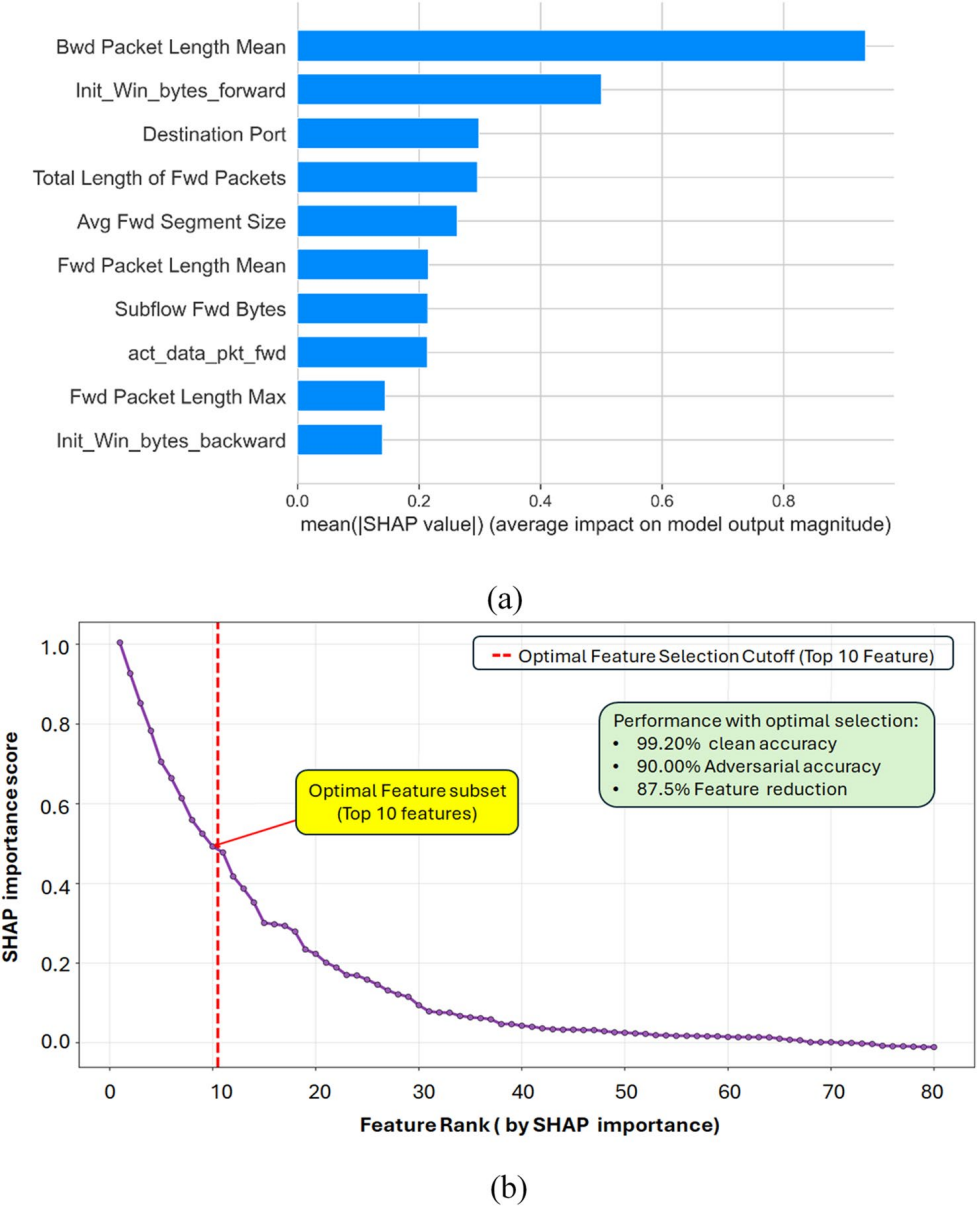
The 10 most important features according to the SHAP method. (**a**) The Top 10 Feature importance. (**b**) The SHAP Optimal Feature subset.

The SHAP feature importance analysis identified the ten most influential predictors driving the CatBoost model’s DDoS detection performance, as shown in Fig. 4(a). The SHAP ranking curve further revealed a clear inflection at the tenth feature, establishing the optimal selection cutoff, illustrated in Fig. 4(b). The impact of this SHAP-guided reduction was evaluated through a systematic ablation study. Using the optimized 10-feature subset, the full OCML framework achieved 99.20% clean accuracy and 90.00% adversarial accuracy. To isolate the effect of feature selection, a comparative model was trained without SHAP (Without_SHAP), retaining all 80 features. Although this model exhibited improvements over the unoptimized baseline (+ 5.09% clean accuracy; +28.07% adversarial accuracy), it demonstrated lower stability, reflected in a robustness drop of 0.2230—substantially larger than that observed in OCML. These results confirm that SHAP not only provides effective dimensionality reduction but also enhances decision consistency and robustness by concentrating learning on the most informative and least noisy features.

**Fig. 5:**
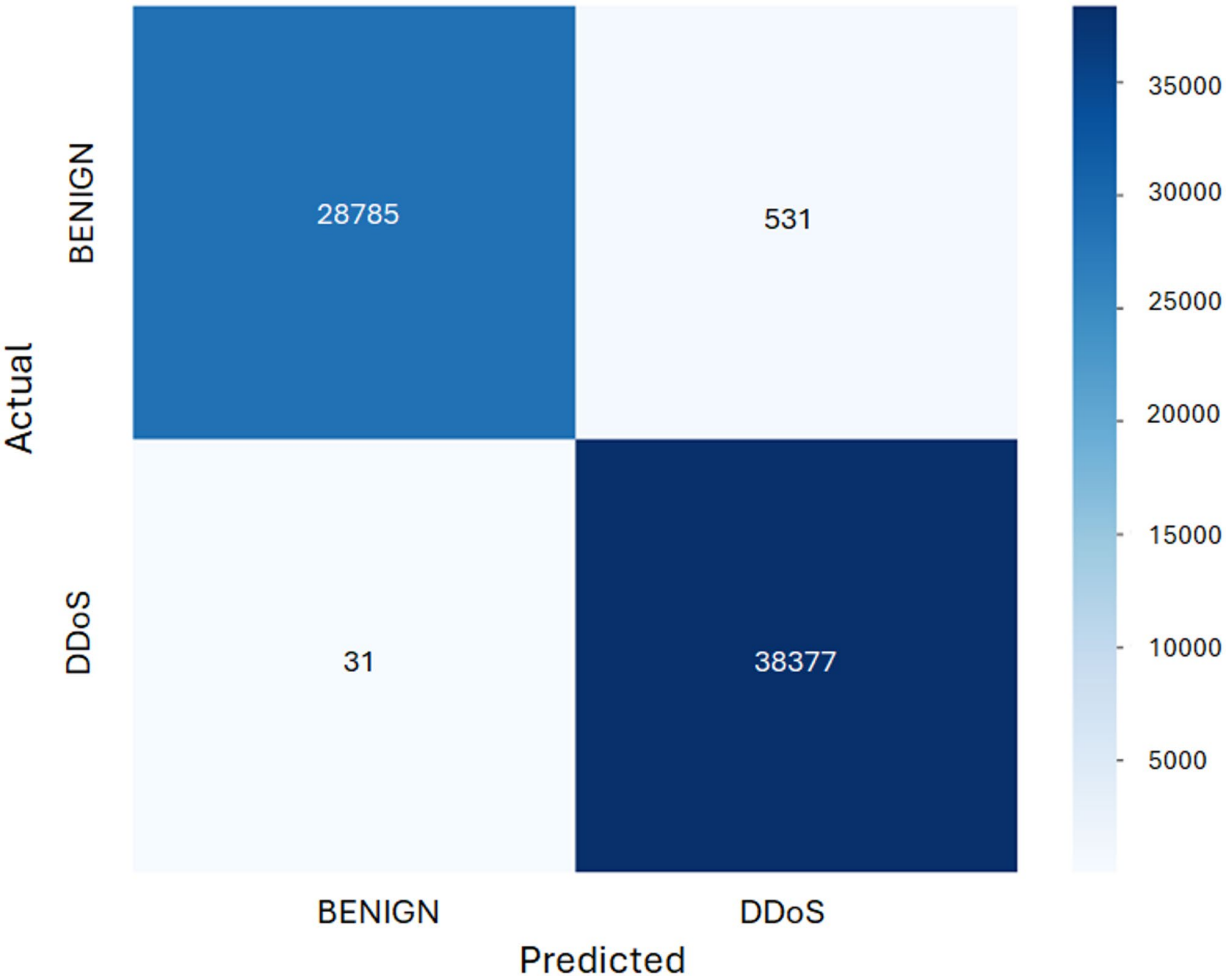
The confusion matrix of the proposed model.

Figure 5 presents the classification performance of the model in distinguishing between benign (class 0) and DDoS attack (class 1) traffic. Shows that the model achieves high classification accuracy, correctly identifying 38,771 DDoS instances and 27,886 benign samples, with only minor misclassifications (17 false negatives and 501 false positives).

## Case studies

**Fig. 6:**
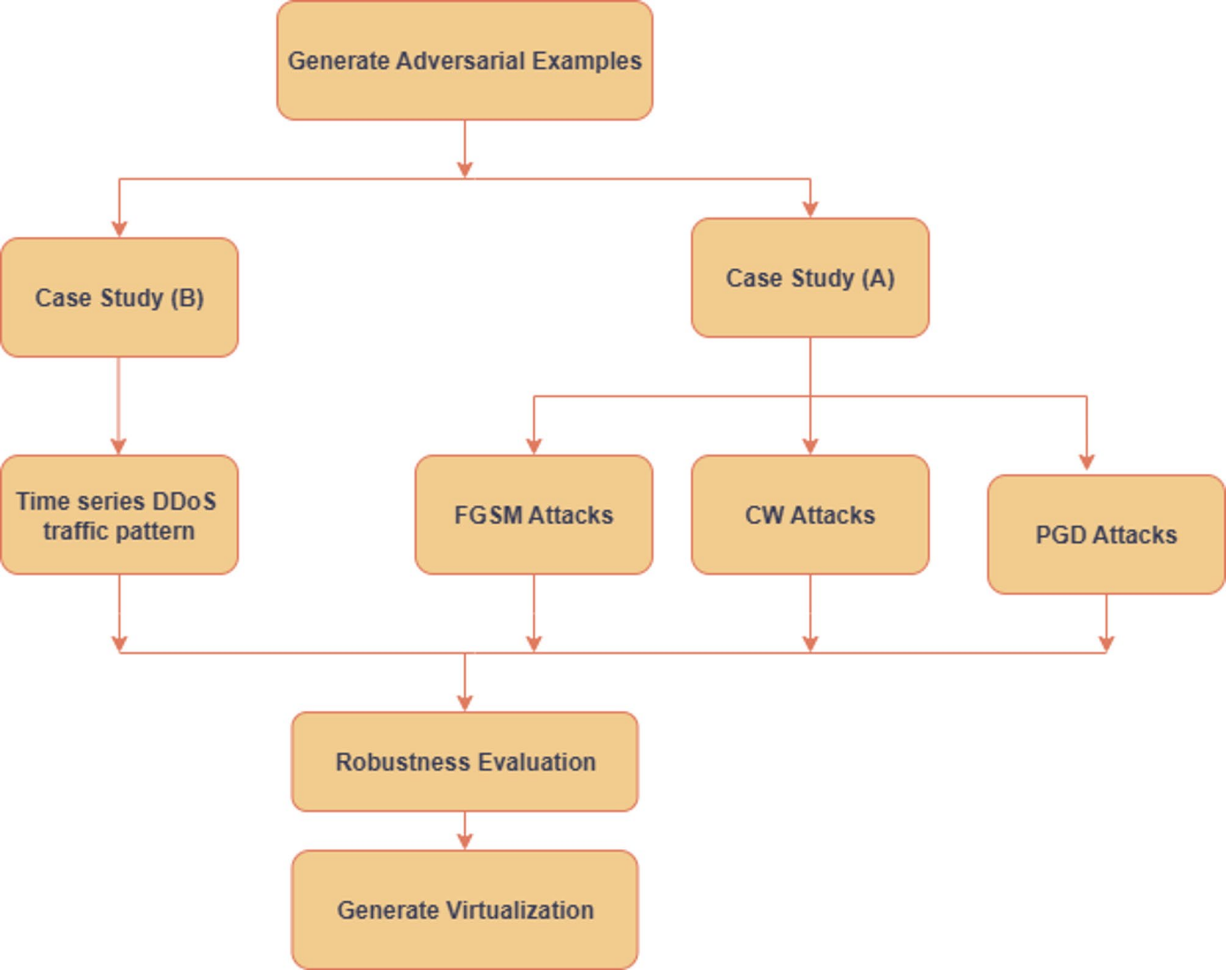
Case studies of the proposed framework.

In this section, two case studies are presented to evaluate the robustness of the proposed model. Case Study A involves the implementation of adversarial attack techniques, specifically the Fast Gradient Method (FGSM), the Carlini-Wagner (CW), and Projected Gradient Descent (PGD) attacks, to assess the model’s performance under adversarial conditions. The outcomes are compared using test data, and key performance metrics are computed. Case Study B focuses on evaluating the model in a dynamic environment by simulating variations in DDoS attack patterns over time. This scenario assesses the model’s adaptability and effectiveness in detecting evolving attack behaviors. Figure 6 illustrates the experimental setup and methodology for both case studies.

### Case study (A)

**Fig. 7:**
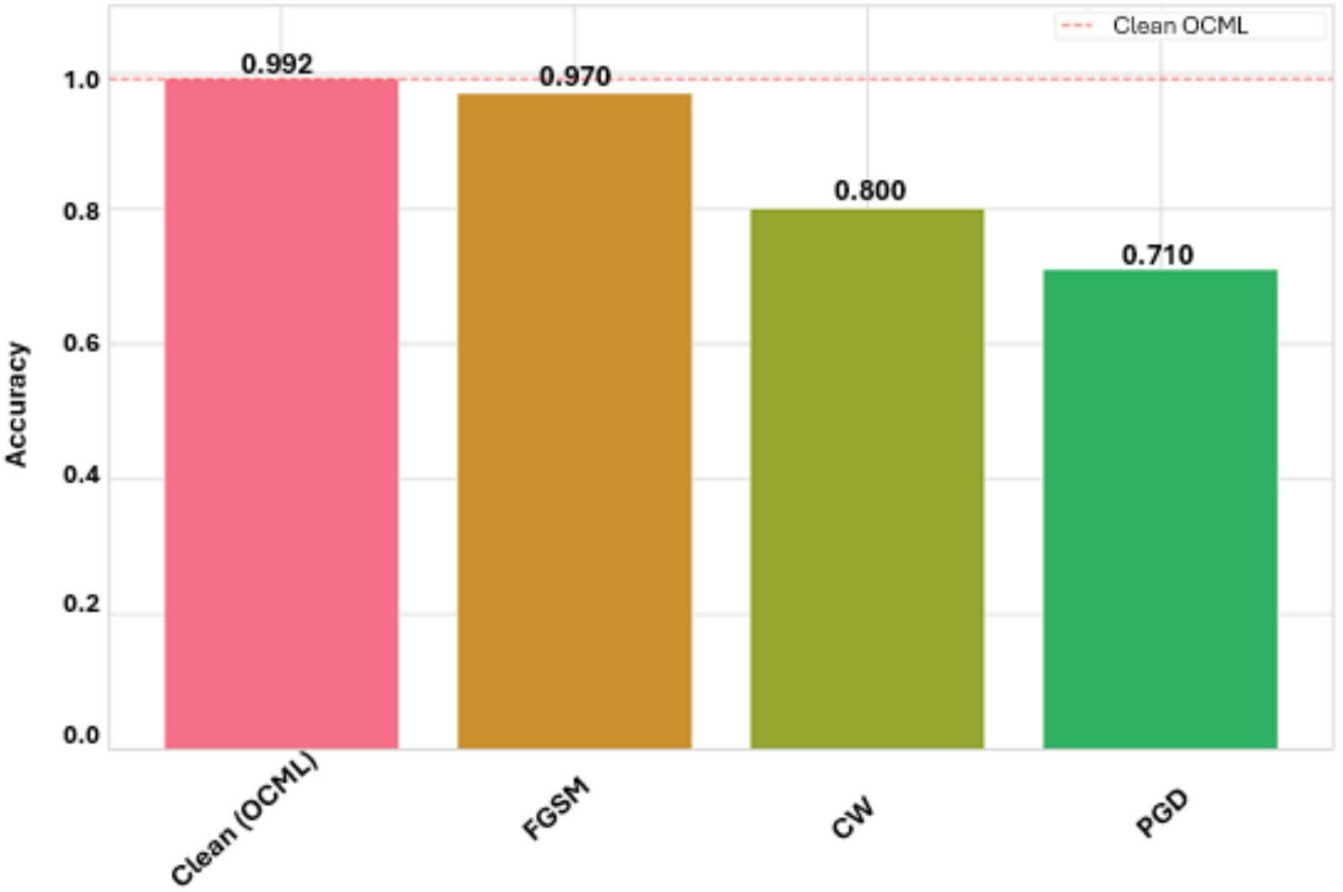
Model accuracy under adversarial attack.

The evaluation of the adversarially trained model yielded a clear and interpretable hierarchy of performance against the selected white-box attacks, as depicted in Fig. 7. The model achieved a baseline accuracy of 99.2% on benign, unperturbed data. As expected, this represents the upper bound of performance, with a marginal decrease from a standardly trained model, illustrating the inherent trade-off between nominal accuracy and adversarial robustness.

When subjected to adversarial perturbations, the model demonstrated strong resilience to the FGSM attack, maintaining an accuracy of 97%. This result indicates that training against a strong, iterative adversary like PGD confers robust protection against weaker, single-step gradient-based threats. The accuracy against the more sophisticated C&W attack was measured at 80%. This substantial, yet not complete, degradation in performance is a critical finding; it suggests that the model has learned genuinely robust features rather than relying on obfuscated gradients, as the C&W attack is specifically designed to bypass such naive defenses.

Finally, the model’s accuracy against the strong 40-iteration PGD attack was 71%. This being the lowest accuracy score is the most direct confirmation of the training’s success. It establishes the model’s performance floor against the very threat it was optimized to resist, demonstrating that while not invulnerable, the model possesses a significant and measurable level of robustness. The ordered decline in accuracy from Clean to FGSM, C&W, and finally PGD aligns perfectly with theoretical expectations, confirming that the PGD adversarial training regimen successfully fortified the model against white-box threats. All attack evaluation summaries are presented in Table 4.

**Table 4:** Comparison between clean data and adversarial attacks.

Attack	Accuracy	Success rate	DDOS F1	Benign F1	Robustness
Clean (OCML)	0.9917	–	0.9927	0.9903	0.9917
FGSM	0.9700	0.0050	0.9697	0.9703	0.9775
C&W	0.8000	0.0250	0.8012	0.8413	0.8011
PGD	0.7100	0.3150	0.5797	0.7786	0.7025

**Fig. 8:**
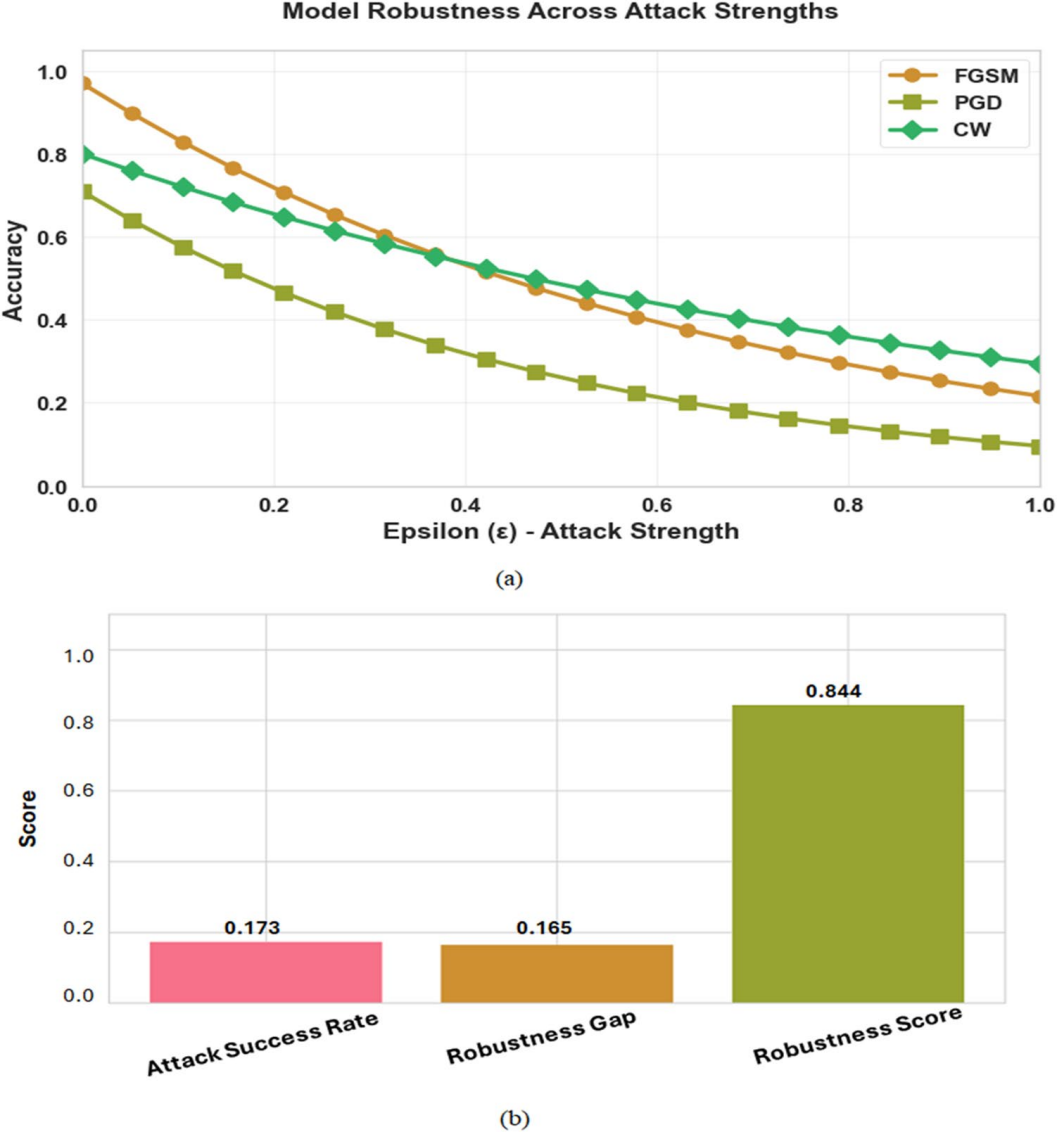
(**a**) Model robustness cross attack strengths (**b**) Model robustness metrics summary.

To comprehensively assess the model’s resilience, its performance was evaluated across a continuous spectrum of attack strengths, measured by the perturbation budget, epsilon (ε). The results, illustrated in the robustness curve in Fig. 8 (a), reveal a distinct and expected pattern of performance degradation corresponding to the potency of the adversarial method.

Beyond the visual analysis of the robustness curve presented in Fig. 8 (b), the model’s overall resilience was quantified using a set of aggregate metrics. It achieved a low Attack Success Rate (ASR) of 0.173, meaning only 17.3% of attacks were effective. The high Robustness Score of 0.844 confirms strong overall resilience, while the small Robustness Gap of 0.165 indicates minimal accuracy loss under attack. These results show that adversarial training effectively enhanced robustness with little compromise to performance on clean data.

### Case study (B)

**Fig. 9:**
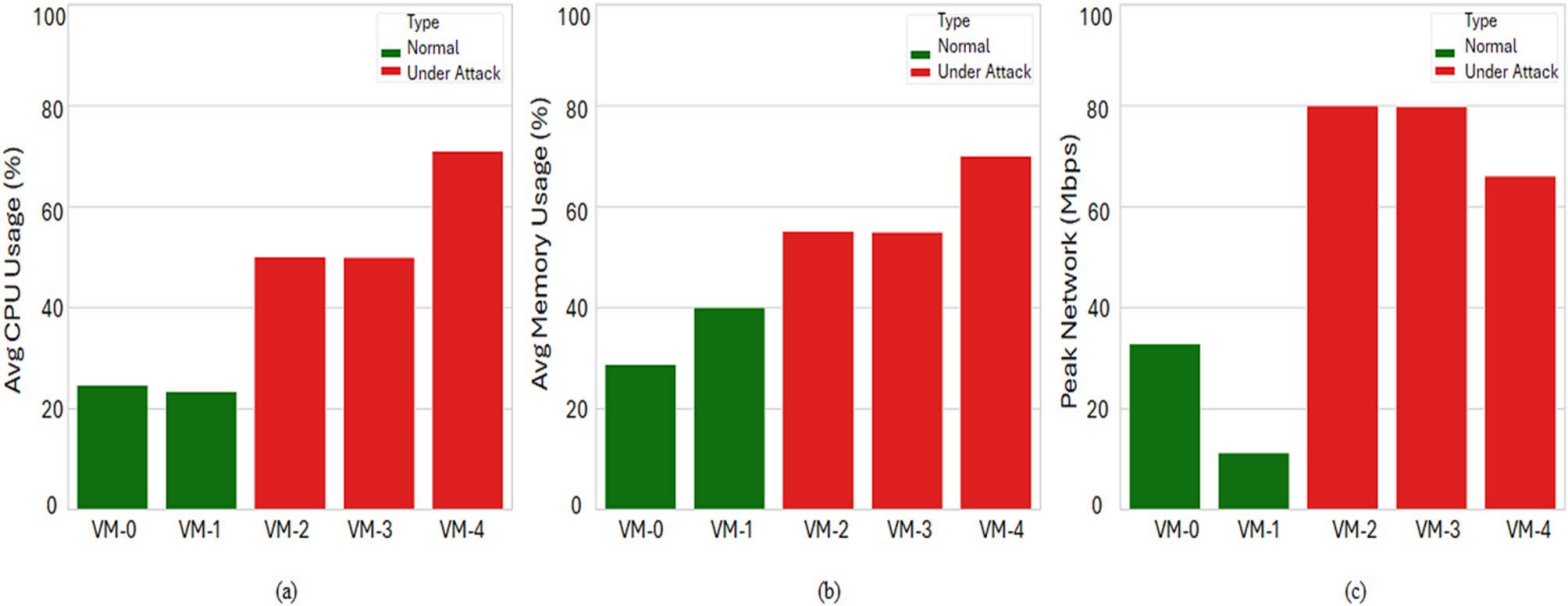
The impact of dynamic traffic spikes on VMs.

The presented Fig. 9 illustrates the impact of traffic spikes, particularly during DDoS attacks, on the performance of virtual machines (VMs) by comparing three key metrics under normal and attack conditions: average CPU usage, average memory usage, and peak network traffic.

Under attack conditions, there is a significant rise in CPU usage across affected VMs. While VM-0 and VM-1 operate under normal load (25% and 23% CPU usage, respectively), VM-2, VM-3, and VM-4 show elevated usage reaching up to 70%, indicating resource strain caused by attack-induced traffic. Similarly, memory usage increases markedly in attacked VMs. VM-0 and VM-1 maintain moderate usage under normal conditions (30% and 40%), whereas VM-2 through VM-4 demonstrate higher memory consumption, with VM-4 peaking near 70%. This rise reflects the additional memory load required to process and buffer excessive traffic. The most significant impact is observed during peak network traffic, where normal VMs (VM-0 and VM-1) exhibit modest usage of under 35 Mbps, while the attacked VMs (VM-2 to VM-4) peak at 65–80 Mbps. This sharp contrast confirms that DDoS attacks induce abnormal traffic volumes that overwhelm VM network interfaces.

The results indicate that DDoS attacks significantly degrade VM performance by increasing CPU and memory utilization, as well as saturating network bandwidth. These metrics serve as critical indicators for real-time detection and response systems in cloud environments.

**Fig. 10:**
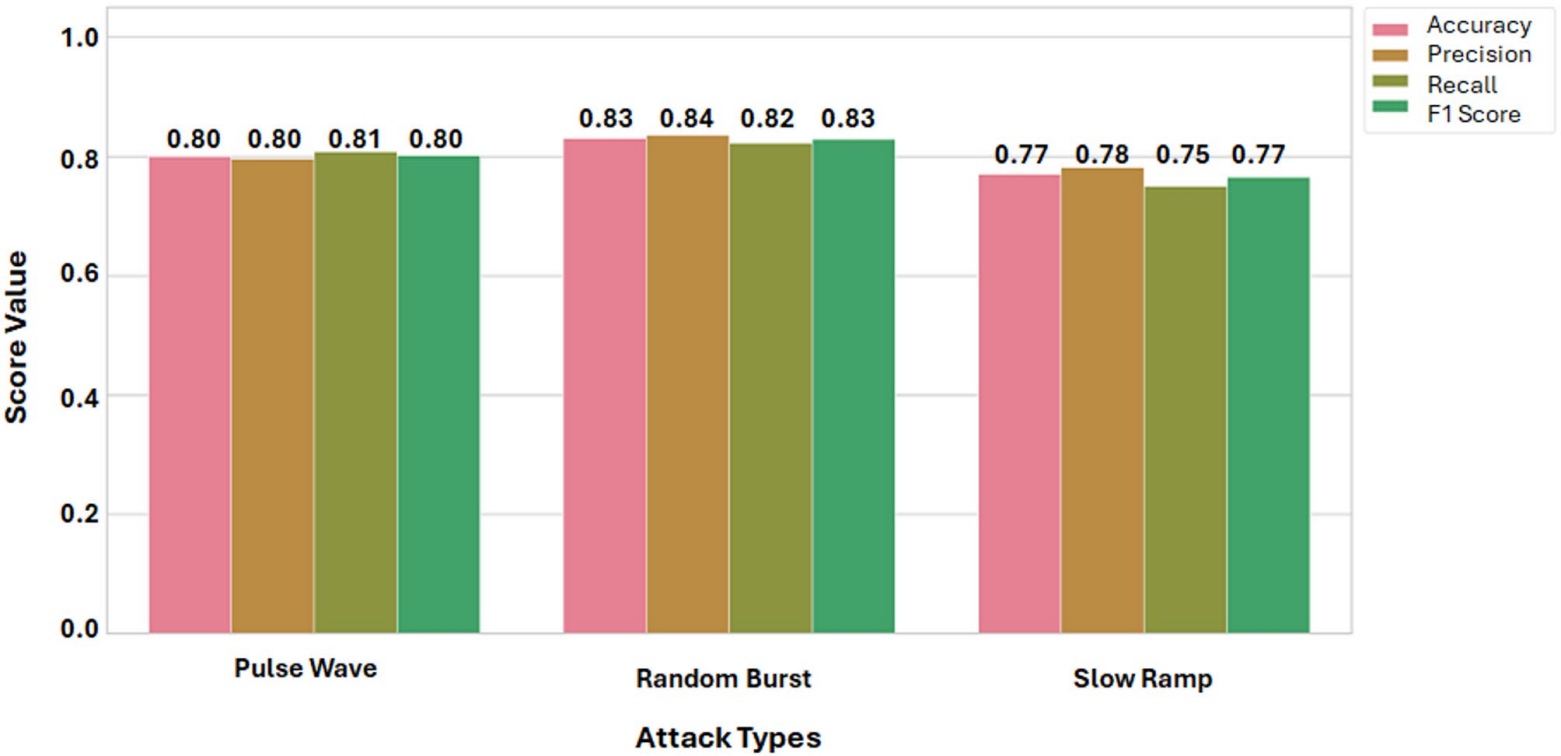
Model detection performance against time series attacks.

The Catboost classifier demonstrated strong overall performance in detecting various DDoS attack patterns presented in Fig. 10, with particularly high accuracy in identifying Random Burst attacks. It achieved an F1-score of 0.83, supported by a precision of 0.84 and a recall of 0.82, indicating effective recognition of erratic, high-variance traffic. For Pulse Wave attacks, the model maintained a balanced and robust performance, achieving an F1-score of 0.80, with precision and recall both around 0.80. This suggests the model accurately captured the periodic characteristics of the attack while minimizing false positives. Detection of the Slow Ramp attack, known for its subtle behavior, was moderately successful. The model reached an F1-score of 0.77, with a precision of 0.78 and a recall of 0.75, showing that it could still recognize gradual traffic increases despite the attack’s evasive nature.

### Ablation study and component contribution

#### Effect of feature selection (SHAP)

Removing SHAP feature selection (Without_SHAP) resulted in moderate gains over the baseline in both clean (+ 5.09%) and adversarial accuracy (+ 28.07%). Despite using the full 80-feature set, the model remained less stable than OCML, exhibiting a robustness drop of 0.2230. These findings confirm that SHAP not only reduces dimensionality (from 80 to 10 features) but also improves decision consistency and robustness.

#### Effect of hyperparameter optimization (Optuna)

The Without_Optuna model outperformed the baseline in clean accuracy (+ 2.78%) but suffered substantial degradation under adversarial settings (Adv Acc = 0.4900). This indicates that hyperparameter tuning plays a critical role in learning robust decision boundaries. The robustness drop (0.4420) was the highest among all variants, demonstrating the sensitivity of CatBoost to suboptimal hyperparameters.

#### Full OCML framework

The Full_OCML configuration achieved the best performance across all metrics. Table 5 summarizes the differences between model variants.

**Table 5:** Ablation study results for different model variants.

Model	Clean accuracy	Adversarial training accuracy	Robustness drop	Improvement vs. baseline	Features used	Robustness Ratio
Baseline	0.9068	0.5700	0.3368	–	80	0.6286
Without SHAP	0.9530	0.7300	0.2230	0.0462	80	0.7600
Without Optuna	0.9320	0.4900	0.4420	0.0252	10	0.5258
**OCML**	**0.9920**	**0.9000**	**0.0920**	**0.0852**	**10**	**0.9073**

**Fig. 11:**
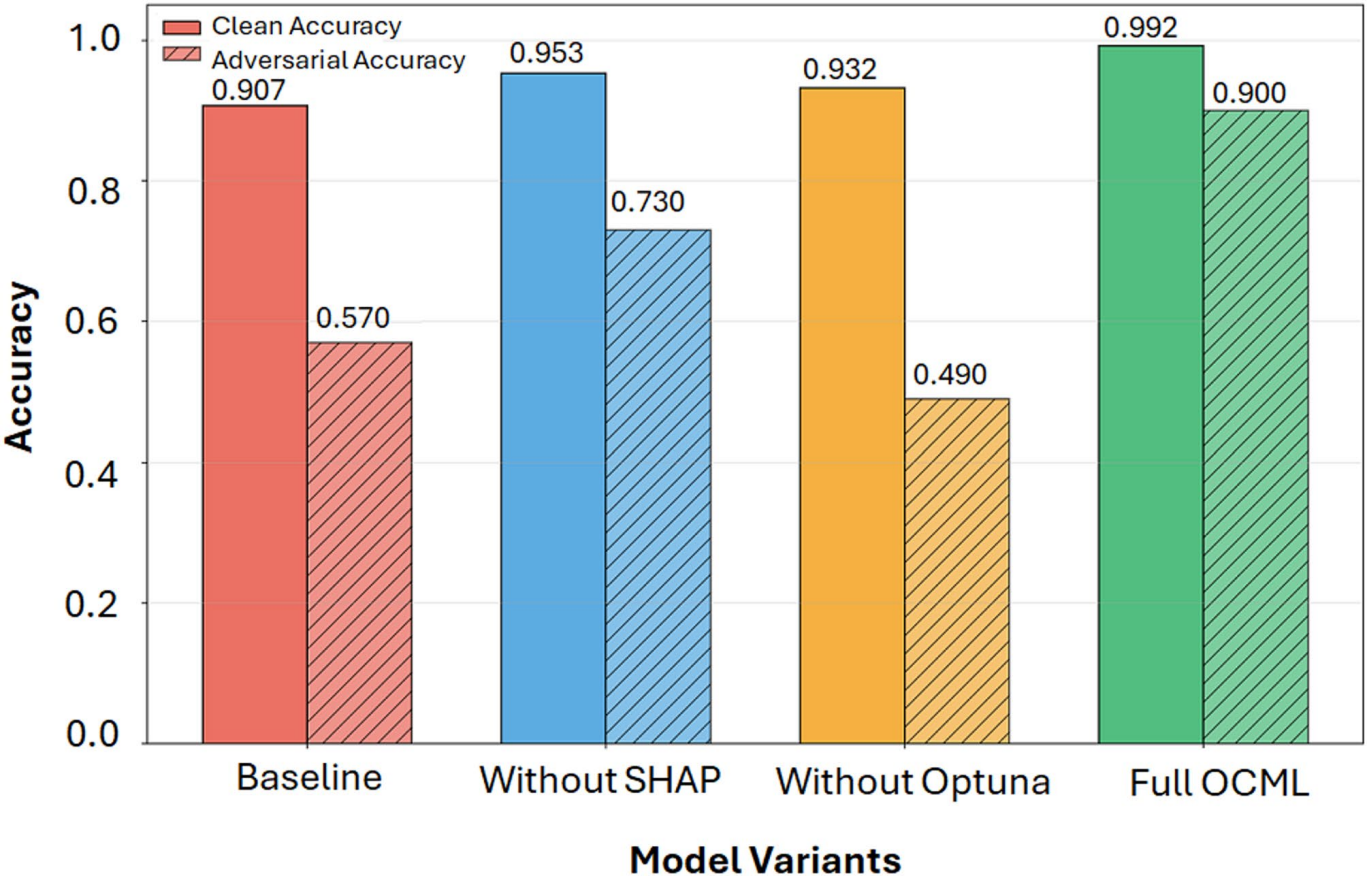
Clean vs. adversarial accuracies across model variants in the ablation study.

Compared to the baseline, OCML improved adversarial accuracy by + 57.89% while simultaneously reducing the feature set by 70 features, demonstrating superior robustness, accuracy efficiency. Figure 11 shows clean vs. adversarial accuracies across model variants.

### Statistical validation of component contributions

The statistical analysis demonstrated that the Full_OCML model significantly outperformed all ablated variants across Clean Accuracy, Adversarial Accuracy, and Robustness Drop. As illustrated in Table 6. One-Way ANOVA indicated highly significant effects of model variant on Clean Accuracy (F = 26.21, *p* < 0.001, η^2^ = 0.019), Adversarial Accuracy (F = 168.39, *p* < 0.001, η^2^ = 0.112), and Robustness Drop (F = 124.28, *p* < 0.001). Tukey HSD confirmed that Full_OCML achieved significantly higher Clean and Adversarial Accuracy and a significantly smaller Robustness Drop than all other models (*p* < 0.05 for all comparisons). Effect size analysis further showed medium to large Cohen’s d values, indicating substantial practical improvements. Full_OCML also achieved a 72.7% reduction in performance degradation under attack, underscoring the practical value of integrating SHAP and Optuna.

**Table 6:** Effect-size calculations further demonstrate the practical impact of the OCML enhancements.

Comparison	Advantage	Cohen’s d	Effect size	Statistical significance
OCML vs. baseline	+ 0.3300	0.806	Large	Significant
OCML vs. without_optuna	+ 0.4100	0.994	Large	Significant
OCML vs. without_SHAP	+ 0.1700	0.448	Medium	Significant

## Discussion

The proposed OCML framework was benchmarked against an extended set of baseline models to rigorously assess its performance within the context of recent advancements in adversarially robust intrusion-detection research. In addition to conventional gradient-boosting classifiers such as CatBoost, XGBoost, and LightGBM^12,18,41^, the evaluation incorporated state-of-the-art adversarial defense techniques reported in contemporary studies, as summarized in Table 7.

Prior work consistently demonstrates that deep-learning-based IDS models—particularly CNN-driven and cloud-oriented architectures—exhibit marked performance degradation under adversarial perturbations^33,34^. In contrast, the OCML framework maintains substantially higher robustness, achieving 97% accuracy under FGSM, 80% under C&W, and 71% under PGD, while retaining a strong clean-traffic baseline of 99.2%. These results indicate that OCML offers both competitive detection accuracy and improved resistance to adaptive adversarial threats and dynamic DDoS traffic patterns, thereby addressing critical limitations in current IDS methodologies.

The proposed optimization framework should be evaluated in real-world cloud environments such as AWS or GCP to validate its scalability and effectiveness. However, such implementation requires substantial computational resources, time, and cost. Despite these challenges, real-world testing would provide valuable insights into its practical PERFORMANCE and robustness.

**Table 7:** Comparative analysis of the proposed OCML and other models in literature.

Model/framework	Dataset used	Adversarial evaluation	Time-series modeling	Key findings/performance
Proposed OCML	CIC-DDoS2019	FGSM, C&W, PGD + adversarial training	Yes — pulse, burst, slow-ramp patterns	99.2% clean accuracy; 97% (FGSM), 80% (C&W), 71% (PGD)
CatBoost^18,41^	CIC-IDS2017	–	–	98–99% clean accuracy
XGBoost^12,18^	CIC-IDS2017 / CERT	–	–	88–99.7% clean accuracy
LightGBM^12,18^	CIC-IDS2017	–	–	97–98% clean accuracy
Optimized ANN^26^ Hybrid SVM^27^ DLNN & LSTM^28^	UNSW-NB15 NSL-KDD	–	–	99.4% clean accuracy 97% 99.53% & 98,53%
CNN with adversarial training^33^	MNIST	FGSM, CW	–	98.89% preserving accuracy
Cloud IDS with adversarial training + SHAP feature selection^34^	CSE-CIC-IDS2018	FGSM, PGD	–	60% FGSM and 55% PGD accuracy

## Conclusion and future work

The prevalence of malicious activities in cloud computing environments is continuously increasing, with data security remaining a primary concern. This study presented an optimized machine learning framework (OCML) that integrates CatBoost classification, SHAP-based feature selection, and adversarial robustness enhancement for detecting Distributed Denial-of-Service (DDoS) attacks in cloud virtual machine environments. By reducing the feature space using SHAP, the model was trained on a more informative subset of attributes, contributing to stable classification performance. The adversarial evaluation showed that PGD-based adversarial training improved the resilience of the classifier, yielding an accuracy of 84.4% under three strong white-box attack scenarios and reducing the overall Attack Success Rate to 17.3%. When applied to DDoS detection using the CIC-DDoS2019 dataset, the optimized CatBoost model achieved F1-scores ranging from 0.77 to 0.83 across multiple attack categories. These results indicate that combining feature optimization with adversarial training can enhance both predictive accuracy and robustness within the evaluated experimental setting.

Although the framework demonstrates promising performance, its generalizability remains constrained by dataset-specific characteristics and the simulated nature of the adversarial attacks. Additionally, the evaluation does not capture the full variability of real-world cloud traffic, which may exhibit higher complexity and noise.

Future research will focus on evaluating the OCML framework under more diverse, dynamic, and high-volume traffic conditions to better assess its generalization capabilities. Testing the system in operational cloud environments—such as AWS, Azure, or GCP—will be essential for examining scalability, latency, and end-to-end operational feasibility. Further extensions may include comparative analysis with additional gradient-boosting architectures, exploration of online or adaptive learning strategies, and integration with real-time monitoring systems for continuous threat detection.

## Data Availability

The data generated or analyzed during this study are included in this published article and for requesting any data contact the corresponding author Hadder Samy (mailto: hadeersamy1998@gmail.com).
